# Electrical Stimulation Using Conductive Polymer Polypyrrole Counters Reduced Neurite Outgrowth of Primary Prefrontal Cortical Neurons from NRG1-KO and DISC1-LI Mice

**DOI:** 10.1038/srep42525

**Published:** 2017-02-15

**Authors:** Qingsheng Zhang, Dorna Esrafilzadeh, Jeremy M. Crook, Robert Kapsa, Elise M. Stewart, Eva Tomaskovic-Crook, Gordon G. Wallace, Xu-Feng Huang

**Affiliations:** 1 Centre for Translational Neuroscience, School of Medicine, University of Wollongong, Wollongong, NSW 2522, Australia; 2 ARC Centre of Excellence for Electromaterials Science, Intelligent Polymer Research Institute, AIIM Facility, Innovation Campus, University of Wollongong, Squires Way, Fairy Meadow, NSW 2519, Australia; 3 Illawarra Health and Medical Research Institute, Wollongong, NSW 2522, Australia; 4 Departments of Surgery, St Vincent’s Hospital, The University of Melbourne, Fitzroy, VIC 3065, Australia; 5 Departments of Medicine, St Vincent’s Hospital, The University of Melbourne, Fitzroy, VIC 3065, Australia; 6 Schizophrenia Research Institute, 384 Victoria Street, Darlinghurst, NSW 2010, Australia

## Abstract

Deficits in neurite outgrowth, possibly involving dysregulation of risk genes neuregulin-1 (NRG1) and disrupted in schizophrenia 1 (DISC1) have been implicated in psychiatric disorders including schizophrenia. Electrical stimulation using conductive polymers has been shown to stimulate neurite outgrowth of differentiating human neural stem cells. This study investigated the use of the electroactive conductive polymer polypyrrole (Ppy) to counter impaired neurite outgrowth of primary pre-frontal cortical (PFC) neurons from NRG1-knock out (NRG1-KO) and DISC1-locus impairment (DISC1-LI) mice. Whereas NRG1-KO and DISC1-LI exhibited reduced neurite length and number of neurite branches compared to wild-type controls, this was not apparent for cultures on electroactive Ppy. Additionally, the use of the Ppy substrate normalised the synaptophysin and PSD95 protein and mRNA expression whereas both are usually reduced by NRG1-KO or DISC1-LI. Our findings support the utility of Ppy mediated electrical stimulation to prevent the reduction of neurite outgrowth and related synaptic protein expression in the primary PFC neurons from NRG1-KO and DISC1-LI mice, providing proof-of-concept for treating neurodevelopmental diseases including schizophrenia.

Schizophrenia is a debilitating mental disorder affecting over 1% of the world’s population. Current treatment relies mainly on antipsychotic drugs that have limited efficacy for treating cognitive dysfunction, a core feature of schizophrenia. There is an urgent need for alternative therapies^1^, with deficits in neurodevelopment involving neurite outgrowth and function being potential targets^2^.

NRG1 and DISC1 are important in brain development and plasticity^3^,^4^, as well as being recognised susceptibility genes in schizophrenia. Mice with heterozygous deletion of NRG1 have impaired cognitive function in pre-pulse inhibition, latent inhibition^5^,^6^,^7^, and social interaction behaviour^8^,^9^. Moreover, the role of NRG1 in regulating prefrontal cortical neurite outgrowth has recently been suggested^3^,^10^. Similarly, defects in the DISC1 gene have been reported to reduce neurite outgrowth, spine density and synaptic plasticity^2^,^11^,^12^,^13^,^14^,^15^.

Ppy is a conducting polymer used in biosensors, drug delivery and neural prosthetics^16^,^17^,^18^,^19^. We have previously shown that the electroactive Ppy doped with Dodecylbenzenesulfonic acid (DBSA) promotes neuronal induction including neurite outgrowth and branching of differentiating frontal cortical human neural stem cells^18^. Moreover, earlier studies of primary rat spiral ganglion neurons show that Ppy-DBSA is cytocompatible and supports greater neurite outgrowth compared to Ppy with other dopants^20^. Ppy-DBSA combined with electrical stimulation may therefore correct impaired neurite growth arising from gene dysregulation relevant to schizophrenia, such as NRG1 and DISC1 haploinsufficiency. Here we present supporting evidence of an effect of electroactive Ppy-DBSA on neurite outgrowth and synaptic protein expression in primary prefrontal cortical (PFC) neurons from heterozygous NRG1 transmembrane domain knockout (NRG1-KO) and DISC1 locus impairment (DISC1-LI) mice. Based on our findings we proposed Ppy-DBSA with electrical stimulation has the potential to be used as a new treatment platform for complex neurodevelopmental diseases such as schizophrenia.

## Results

### Neurite outgrowth is improved by Ppy-DBSA with electrical simulation in primary PFC neuronal cultures from wild-type mice

**Figure 1:**
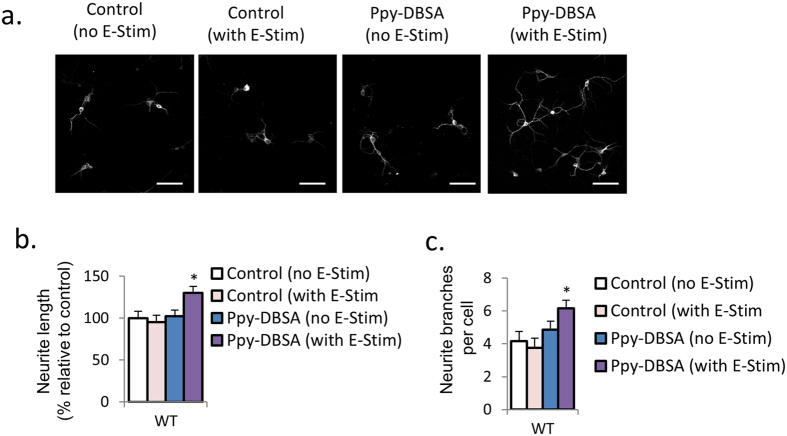
Ppy-DBSA with electrical stimulation improved neurite growth in primary PFC neuronal cultures from wild-type mice. (**a**–**c**) Immunofluorescence with MAP2 reveals that Ppy-DBSA with electrical stimulation upregulates neurite length and the number of neurite branches in mice primary PFC neuronal cultures. Scale bar = 50 μm. Error bars indicate SEM. *p < 0.05 vs control.

Image based analysis of neuronal marker microtubule-associated protein 2 (MAP2) indicated that wild-type PFC neurons cultured on Ppy-DBSA with electrical stimulation have longer neurites (30.2 ± 7.6%, *P* < 0.05) and greater neurite branching (6.2 ± 0.5 vs. 4.2 ± 0.6), with neither apparent for cultures on unstimulated Ppy-DBSA nor stimulated vehicle control (gold mylar) (Fig. 1a–c).

### Neurite outgrowth is supressed in primary PFC neuronal cultures from NRG1-KO and DISC1-LI mice

**Figure 2:**
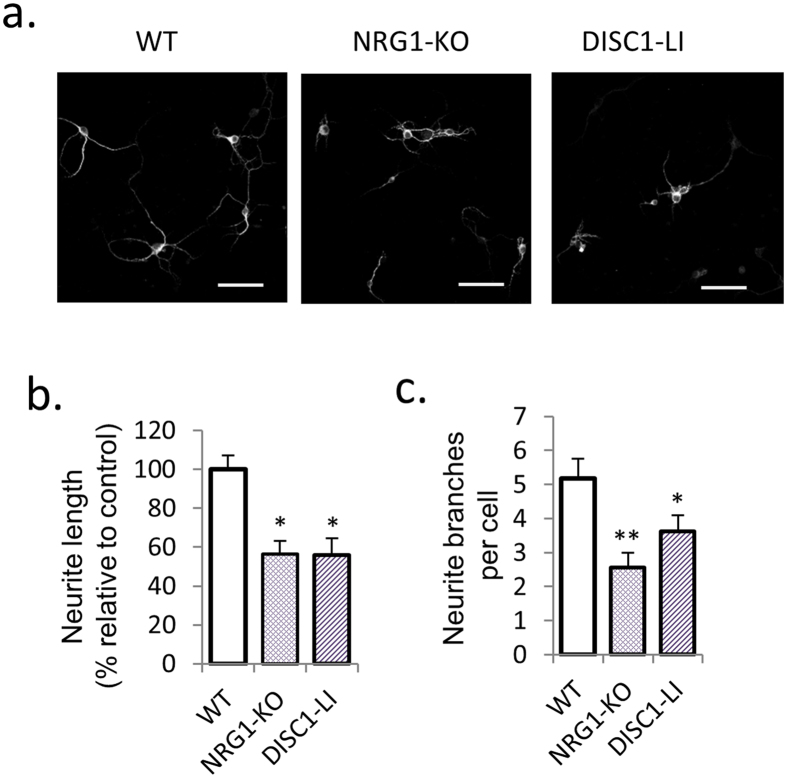
NRG1-KO or DISC1-LI inhibits neurite outgrowth in mice primary PFC neuronal cultures. (**a**–**c**) Neurite length and the number of neurite branches are reduced in PFC primary neuronal cultures from NRG1-KO and DISC1-LI mice, compared to wild-type. Scale bar = 50 μm. *p < 0.05 vs wild-type; **p < 0.01 vs wild-type. Error bars indicate SEM.

We then examined neurite outgrowth in primary PFC neuronal cultures from NRG1-KO and DISC1-LI mice. Compared to wild-type controls, NRG1-KO and DISC1-LI cultures exhibited reduced neurite length (43.3 ± 6.5% and 44.2 ± 8.8% respectively; *P* < 0.05; Fig. 2a,b) and number of neurite branches (50.6 ± 8.5% and 29.9 ± 8.9% respectively; *P* < 0.05; Fig. 2a,c).

### Protein and mRNA expressions of synaptophysin and PSD95 are downregulated in PFC neuronal cultures from NRG1-KO and DISC1-LI mice

Protein and mRNA expression of presynaptic synaptophysin and postsynaptic PSD95 were reduced in the PFC neurons from NRG-KO and DISC1-LI mice. Quantitative western blotting revealed that synaptophysin protein expression decreased by 28.9 ± 6.7% (*P* < 0.05) and 36.5 ± 8.3% (*P* < 0.05) in NRG1-KO and DISC1-LI respectively (Supplementary Figure S1a,b), while PSD95 protein expression was reduced by 31.5 ± 8.4% (*P* < 0.05) and 25.9 ± 7.5% (*P* < 0.05) respectively (Supplementary Figure S1a,c). Quantitative real-time PCR (qRT-PCR) revealed synaptophysin mRNA expression was reduced by 24.9 ± 11.8% (*P* < 0.05) and 34.9 ± 6.2% (*P* < 0.01) in PFC neurons from NRG1-KO and DISC1-LI mice, respectively (Supplementary Figure S1d), and PSD95 mRNA expression was similarly downregulated by 31.4 ± 14.4% (*P* < 0.05) and 27.6 ± 8.6% (*P* < 0.05) respectively (Supplementary Figure S1e); consistent with protein studies.

### Effect of NRG1-KO and DISC1-LI on NRG1 and DISC1 protein and mRNA expression in primary PFC cultures

Quantitative western blotting demonstrated decreased NRG1 protein expression in cultured PFC neurons from NRG1-KO mice (−78.9 ± 7.69%, *P* < 0.001), but not from DISC1-LI mice (Supplementary Figure S2a,b). However, DISC1 protein was reduced in the prefrontal cortical neurons from both NRG1-KO (−36.7 ± 9.39%, *P* < 0.05) and DISC1-LI (−75.9 ± 8.53%, *P* < 0.001) mice (Supplementary Figure S2a,c). Consistent with protein studies, the neurons from NRG1-KO but not DISC1-LI had reduced NRG1 mRNA expression, and neurons from both NRG1-KO and DISC1-LI had reduced DISC1 mRNA expression (Supplementary Figure S2d,e).

### Culture of PFC neurons from NRG1-KO and DISC1-LI mice on electroactive Ppy-DBSA film normalised neurite outgrowth

**Figure 3:**
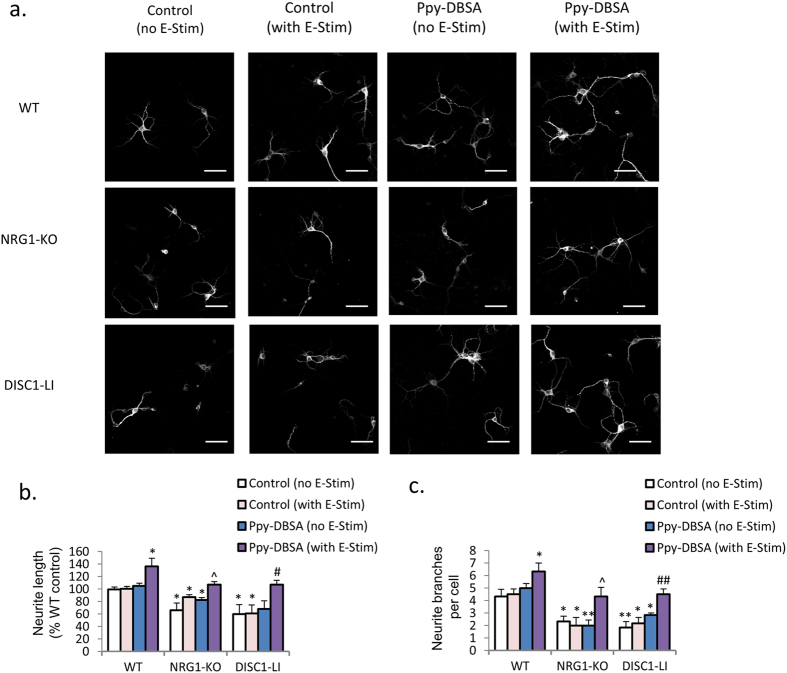
Ppy-DBSA with electrical stimulation improves neurite growth in PFC neuronal cultures from NRG1-KO and DISC1-LI mice. (**a**–**c**) The reduced neurite length and the number of neurite branches are in PFC primary neuronal cultures from NRG1-KO and DISC1-LI mice are improved by Ppy-DBSA with electrical stimulation. Scale bar = 50 μm. *p < 0.05 vs wild-type, control; ^^^p < 0.05 vs NRG1-KO, control; ^#^p < 0.05 vs DISC1-LI, control. Error bars indicate SEM.

Given the effect of Ppy-DBSA with electrical stimulation on neurite outgrowth in primary PFC neurons from wildtype mice, we subsequently investigated NRG-KO and DISC1-LI neurons on electroactive Ppy-DBSA. Mean neurite length and number of neurite branches were comparable between wild-type and mutant neuronal cultures, showing no apparent effect of NRG1-KO and DISC1-LI. In contrast, unstimulated Ppy-DBSA and vehicle control cultures with and without electrical stimulation were characterised by reduced length and branching of neurites (Fig. 3a–c).

### Culture of PFC neurons from NRG1-KO and DISC1-LI mice on electroactive Ppy-DBSA film normalised synaptophysin and PSD95 protein and mRNA expression

Quantitative western blotting indicated increased synaptophysin protein expression (41.2 ± 8.6%, *P* < 0.05) of wild-type neurons cultured on electroactive Ppy-DBSA, while Ppy-DBSA without electrical stimulation or vehicle with electrical stimulation had no effect (Supplementary Figure S3a,b). Whereas PFC cultures from NRG1-KO mice exhibited a decreased synaptophysin protein expression compared to wild type, this was not apparent for cultures on Ppy-DBSA with electrical stimulation (Supplementary Figure S3a,b). A similar effect was found for PFC cultures from DISC1-LI mice (Supplementary Figure S3a,b).

Studies of PSD95 protein demonstrated Ppy-DBSA with electrical stimulation supported an increased expression (39.3 ± 7.3%, *P* < 0.05) compared to vehicle control for PFC cultures from wildtype mice (Supplementary Figure S3a,c). Furthermore, Ppy-DBSA with electrical stimulation prevented the reduction in PSD95 protein expression induced by NRG1-KO or DISC1-LI (Supplementary Figure S3a,c). qRT-PCR revealed a similar trend for mRNA expression of synaptophysin and PSD95 (Supplementary Figure S3d,e).

### Culture of PFC neurons from NRG1-KO and DISC1-LI mice on electroactive Ppy-DBSA film normalised synaptophysin, PSD95, and BDNF immunofluorescence

**Figure 4:**
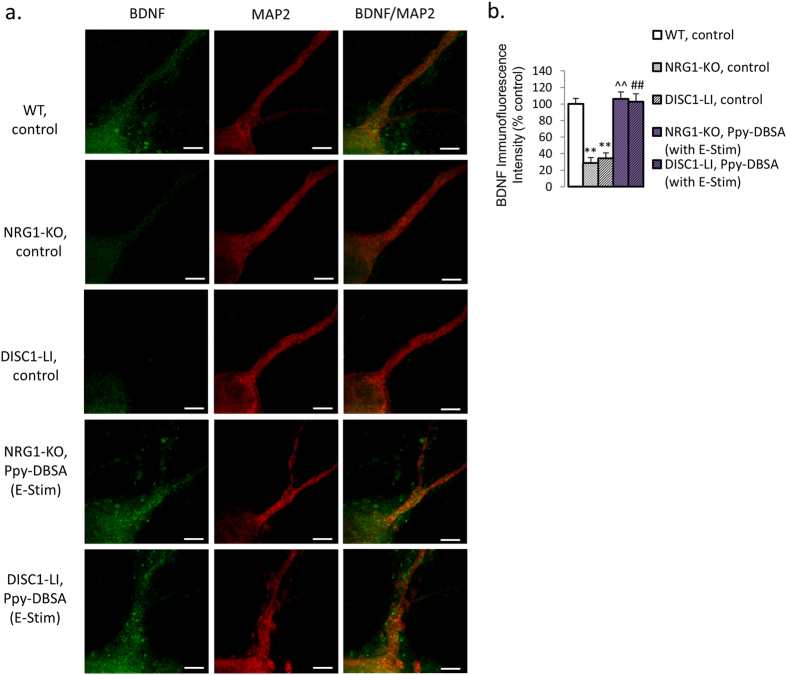
Immunofluorescence of BDNF is reduced in NRG1-KO and DISC1-LI primary PFC neuronal cultures, but rescued by Ppy-DBSA with electrical stimulation. (**a**,**b**) Ppy-DBSA with electrical stimulation reversed the reduced BDNF immunofluorescence induced by NRG1-KO or DISC1-LI, in primary PFC neuronal cultures. Scale bar = 5 μm. *p < 0.05 vs wild-type, control; ^^^^p < 0.01 vs NRG1-KO, control; ^#^p < 0.05 vs DISC1-LI, control. Error bars indicate SEM.

Whereas immunofluorescence intensity of synaptophysin was reduced in neurites from primary PFC neuronal cultures from NRG1-KO and DISC1-LI mice, such reduction was increased by Ppy-DBSA with electrical stimulation (Supplementary Figure S4a,b). Similar effects were found in immunofluorescence of PSD95 (Supplementary Figure S5a,b). These results are consistent with our findings in protein and mRNA studies. Image-based analysis of brain-derived neural factor (BDNF) immunocyotochemistry was reduced for both soma and neurites from primary PFC neuronal cultures from NRG1-KO and DISC1-LI mice, this reduction was prevented by Ppy-DBSA with electrical stimulation (Fig. 4a,b). Protein expression data from westernblot reveals similar results (Supplementary Figure S6a,b).

### Culture of PFC neurons from NRG1-KO and DISC1-LI mice on electroactive Ppy-DBSA film does not normalise NRG1 and DISC1 proteins

To determine whether the effect of electroactive Ppy-DBSA was *via* NRG1 or DISC1 or their downstream targets, quantitative western blotting and qPCR were conducted to examine protein and mRNA levels of NRG1 and DISC1. Electrical stimulation with Ppy-DBSA did not prevent the decreased NRG1 or DISC1 protein/mRNA expression in primary PFC cultures from NRG1-KO or DISC1-LI mice (Supplementary Figure S7a–e).

### Treatment with BDNF improved synaptophysin and PSD95 immunofluorescence and protein expression

Since BDNF levels are elevated by electroactive Ppy-DBSA film, we subsequently tested the hypothesis that exogenous BDNF administration similarly increases synaptophysin and PSD95 expression of primary PFC neuronal cultures. Image-based analysis of synaptophysin and PSD95 showed that exogenous BDNF treatment of PFC neurons cultured from WT, NRG1-KO, and DISC1-LI mice increased synaptophysin and PSD95 protein expression, while exogenous BDNF + antagonist ANA12 had no significant effect, in (Supplementary Figures S8–S13). Similarly, synaptophysin and PSD95 protein expression measured by westernblot was elevated by exogenous BDNF, while co-treatment with antagonist ANA12 completely prevented the effect of BDNF (Supplementary Figure S14).

## Discussion

It has been reported that bioactive conducting polymers, such as polypyrrole, can promote cell attachment^21^,^22^, provide guidance cues for neurite extension^23^,^24^, and deliver effective electrical stimulation^25^,^26^. Here we report the use of Ppy for reversing the supressed neurite outgrowth induced by NRG1 and DISC1 loss-of-function in PFC neuronal cultures. We initially confirmed that the use of the Ppy substrate with electrical stimulation augments neurite growth in primary PFC neuronal cultures from wild-type mice. This is consistent with our previous findings^18^. Moreover, we independently demonstrated supressed neurite outgrowth in NRG1-KO and DISC1-LI mouse PFC neurons, followed by studies of the effect of Ppy with electrical stimulation on primary neurons from the mutant mouse models.

Our findings verify both the use of primary PFC neuronal cultures from NRG1-KO and DISC1-LI mice for phenotypic studies relevant to schizophrenia as well as the potential utility of electroactive Ppy-DBSA as both a research and therapeutic platform for neurite growth deficits in neuropsychiatric disease. Both NRG1 and DISC1 are regarded as key genetic susceptibility factors for neurodevelopmental deficits in schizophrenia and related disorders^2^. In addition, DISC1 is a putative downstream target in the NRG1-ErbB4 signaling pathway^2^,^27^ (presently supported by the effect of NRG1 KO on DISC1 protein and transcript expression in cultured PFC neurons). By targeting NRG1 and DISC1 signalling using implantable, cytocompatible and conducting Ppy for diseased neural tissue interfacing, *in vivo* cell and tissue remodelling may be achievable towards normalisation of tissue function. Given the fact that NRG1 and DISC1 are involved in other mental disorders^2^, the application of the findings of the present study could be extended beyond the initial aim of preventing neurite outgrowth deficits in schizophrenia, to other mental diseases sharing the similar pathological deficits.

Findings of synaptophysin and PSD95 protein and mRNA are consistent with morphological changes. We have previously reported reduced synaptophysin and PSD95 protein and transcript in primary PFC neuronal cultures from NRG1-KO mice^10^. While we have presently shown rescue of the synaptic markers by use of the Ppy substrate, the mechanism for change remains to be determined. However, it could reasonably be argued that marker expression reflects the number of synapses which in turn is impacted on by the number of neurites (provisionally supported by immunofluorescence studies of synaptophysin and PSD95; Supplementary Figures S4 and S5).

The absence of any change in NRG1 or DISC1 protein and transcript expression suggests an effect of the Ppy on neurite growth downstream of NRG1 and DISC1. Interestingly, electrical stimulation via the PPy substrate increased BDNF protein expression (an important neuro-growth cytokine produced downstream of NRG1 and DISC1 signaling pathway), which is reduced in NRG1-KO and DISC1-LI neuronal cultures (Fig. 4; Supplementary Figure S6), suggesting that BDNF may be an important mediator in the effects observed.

We further investigated the effect of exogenous BDNF administration on protein expression and immunofluorescence of synaptophysin and PSD95. Our results showed that exogenous BDNF can mimic the effect of electroactive PPy-DBSA by rescuing the expression of synaptophysin and PSD95 protein of PFC neurons cultured from NRG1 and DISC1 loss-of-function models. This suggests that the effect of electroactive PPy-DBSA may be mediated, at least partially, by the BDNF pathway.

In conclusion, findings from the present study suggest that the use of the PPy substrate and electrical stimulation is effective in preventing the reduction of neurite outgrowth and related expression of synaptic proteins in primary PFC neurons cultured from NRG1-KO and DISC1-LI mice. Considering the clinical relevance of deficits in NRG1 and DISC1 gene function in neurodegenerative and neuropsychiatric disorders (such as schizophrenia, Parkinson’s disease, and Alzheimer’s disease), our findings could be considered proof-of-concept for a new treatment strategy of these diseases.

## Methods and Materials

### Material Preparation

The Pyrrole (Py) monomer and the dopant, dodecyl benzene sulfonic acid (DBSA) were obtained from Sigma. The Py was distilled prior to use. All Py monomer and dopant solutions were prepared with deionized water (Milli-Q). Gold-coated mylar (Solutia Performance Films, CA, USA) was prepared for polymerisation by cleaning with isopropanol and Milli-Q, then dried under a N2 stream. Aqueous monomer solutions were prepared separately for the dopant, consisting of 0.2 M Py and 0.05 M DBS. These aqueous solutions were degassed using N2 for 10 min prior to polymerization of the polymers. PPy films were polymerized galvanostatically at a current density of 0.1 mA/cm^2^ for 10 min in the aqueous monomer solution using an eDAQ EA161 potentiostat. Polymer growth was performed in a standard three-electrode electrochemical cell with the gold-coated mylar as the working electrode, a platinum mesh counter electrode, and an Ag|AgCl reference electrode. After growth, the films were washed extensively with dH2O, gently dried with N2 gas, and stored under desiccated conditions before being prepared for cell culture. Polymers were sterilized by soaking for 20 min in 70% EtOH and then air dried prior to coating with poly-D-lysine (1 h at room temp), then laminin (20 μg/mL, 4 °C overnight; Life Technologies). Solutions were removed and platforms allowed to air dry before cell seeding.

### Primary Cell Cultures

Experiments were run in 6 parallels and repeated for 3 times. The heterozygous NRG1-KO mice^7^ and the DISC1-LI mice^15^,^28^ have been described previously. (The original heterozygous DISC1-LI mice were obtained from Prof Sawa’s group from John Hopkins University.) Heterozygous NRG1-KO male and wild-type C57BL/6 J female mice were mated to obtain either heterozygous NRG1-KO or wildtype pups. Similarly, heterozygous DISC1-LI male and wild-type C57BL/6 J female mice were mated to obtain either heterozygous DISC1-LI or wildtype pups. All experimental procedures were approved by the Animal Ethics Committee, University of Wollongong, Australia, and were complied with the Australian Code of Practice for the Care and Use of Animals for Scientific Purposes^29^.

On postnatal Day 0 (PN0), genotypes were determined by tail biopsy and polymerase chain reaction (For NRG1 mating: primers for Nrg1-KO mice: Neo173F 5′-ATGAACTGCAGGACGAGGCA-3′ and Neo6301R 5′-GCCACAGTCGATGAATCCAG-3′; primers for wild-type mice: 5′-AACAGCCTGACTGTTAACACC-3′ and 5′-TGCTGTCCATCTGCACGAGACTA-3′. For DISC1 mating: primers for DISC1-LI: 5′-TTCCCTTTCTCACCCACACAGG-3′ and 5′-TCCAGATAACTGCCGTCACTCC-3′; primers for wild-type mice: 5′-TTCCCTTTCTCACCCACACAGG-3′ and 5′-CTGGGTGTAGCTAATGGATCCG-3′). Primary pre-frontal cortical cultures were prepared as described previously^30^. In brief, postnatal Day 0 (PN0) pups were decapitated and brains were dissociated aseptically. Brains were then placed into ice cold dissecting solution. After careful removal of the meninges and superficial blood vessels, the prefrontal cortex area from both hemispheres was dissected and cut into approximately 1mm pieces. The tissue pieces were incubated at 37 °C for 30 min in papain-containing enzyme solution. Digested tissue pieces were washed in solutions containing enzyme inhibitors, and then dissociated by triturating in neurobasal media (NBM) through glass pipettes and collected in warm NBM supplemented with B27. Dissociated cortical cells were plated at a final density of 5 × 10^5^cells/cm^2^ on PDL-coated 4-well chambers on gold mylar (Control) or gold mylar with polypyrrole polymers (Ppy-DBSA). 5-FDU were added into the conditional NBM (which was used to replace the NBM) to halt the growth of non-neuronal cells. Cultures were maintained at 37 °C in a humidified CO2 incubator with media changed every 3 days. Cells were used for experiments on 7 days *in vitro* (DIV7).

### Electrical Stimulation

Electrical stimulation was applied to corresponding groups of cell culture in accordance with the clinically relevant published method^25^. Briefly, cells were stimulated for 8 h per 24-h period for 3 days under 5% CO2 humidified atmosphere at 37 °C. Electrical stimulation was performed through a two-electrode setup, whereby an auxiliary platinum mesh electrode was placed into the media at the top of each well and the PPy coated Au-mylar surface was used as the working electrode. The cells were stimulated at ±0.25 mA/cm^2^ using a biphasic waveform of 100 μs pulses with 20 μs interphase open circuit potential and a 3.78 ms short circuit (250 Hz) using a Digital StimulatorDS8000 and A365 Isolator units (World Precision Instruments) interfaced with an e-corder system (eDAQ). The voltage waveform across the active electrode area in response to the current pulse applied was recorded.

### Immunofluorescence and Imaging

Cells were grown on gold mylar films with or without Ppy-DBSA coating until DIV7, when electrical stimulation (8 hours per day, for 3 consecutive days) were conducted (for groups without electrical stimulation, films were placed next to the stimulation groups so that the cells have the same growth conditions as those in the stimulation groups) before being fixed in 4% paraformaldehyde. Cells were then washed in PBS, and permeabilized with 0.3% Triton X-100 in PBS for 10 min. After blocking with 5% normal goat serum for 1 h at room temperature, primary antibody incubations were performed in 1% goat serum in PBS overnight at 4 °C, followed by incubation in a secondary antibody cocktail of Alexa Fluor 488, 594, and DAPI (Life Technologies, NSW) for 2 h at room temperature. Cells were viewed using a 40× or a 63× oil immersion objective on a DMI6500B confocal microscope (Leica, Mannheim, Germany). Image J 1.46r with add-in NeuriteQuant was used for morphological analysis.

### Western Blot

After electrical stimulation, cells were harvested with lysis buffer (containing NP40, Protease Inhibitor Cocktail, 1 mM PMSF and 0.5 mM β-glycerophosphate). Total protein concentrations were determined by DC-Assay (Bio-Rad, Hercules, CA), and detected using a SpectraMax Plus384 absorbance microplate reader (Molecular Devices, Sunnyvale, CA). Samples were heat-treated in Laemmli buffer at 95 °C, loaded to 8% SDS-PAGE gels for fractionation, and then transferred onto Immun-BlotTM PVDF membranes (Bio-Rad, Hercules, CA). The block consists of 5% BSA in TBST. The membranes were then incubated with synaptophysin (Life Technologies, NSW; #701503; dilution factor: 1:1000), PSD95 (Life Technologies, NSW; #516900; dilution factor 1:1000), BDNF (Santa Cruz Biotechnology, Santa Cruz, CA; #sc-20981; dilution factor 1:500), NRG1 (Santa Cruz Biotechnology, Santa Cruz, CA; #sc-348; dilution factor 1:500), or DISC1 (Abcam, Waterloo, NSW; #ab192258; dilution factor 1:1000) antibodies in TBST containing 1% BSA overnight at 4 °C. Mouse anti-actin primary polyclonal antibody (1:10000; Millipore, #MAB1501) was used to determine the actin levels. Secondary antibodies were goat anti-rabbit IgG conjugated with horseradish peroxidase (Santa Cruz Biotechnology, Santa Cruz, CA; #sc-2030; dilution factor 1:5000). For visualization, ECL detection reagents were used and films were exposed on the AGFA CP1000 Tabletop Processor (AGFA Healthcare, Scoresby, VIC). Films were analysed using Quantity One software connected to a GS-690 Imaging Densitometer (Bio-Rad, Hercules, CA).

### Quantitative Real-time PCR (qRT-PCR)

The qRT-PCR protocol was adopted from our previous work^31^,^32^. Briefly, total RNA was extracted using the PureLink RNA extraction kit (Life Technologies, NSW) according to the manufacturer’s protocol. First-strand cDNA was synthesized with the VILO cDNA synthesis kit (Life Technologies, NSW) with 20 μL reaction volume. qRT-PCR was carried out in triplicats using TaqMan Gene Expression Assays (Life Technologies, NSW) on LightCycler480+ (Roche, Penzberg, Germany). The results were normalized to mouse β-actin and GAPDH (cat. no. 4331182; Life Technologies, NSW), and were expressed as folds different from control. The assay identification of the target gene were Mm00436850_m1 (synaptophysin), Mm00492193_m1 (PSD95), Mm01212134_m1 (NRG1), and Mm00558040_g1 (DISC1) (Life Technologies, NSW).

### Statistics

SPSS (version 15; Chicago, IL) was used for statistical analysis. One-way analysis of variance (ANOVA) or two-way ANOVAs with post-hoc Tukey’s tests were performed for multiple comparisons. Data were expressed as mean ± SEM, and *p* < 0.05 was considered statistically significant.

## Additional Information

**How to cite this article**: Zhang, Q. *et al*. Electrical Stimulation Using Conductive Polymer Polypyrrole Counters Reduced Neurite Outgrowth of Primary Prefrontal Cortical Neurons from NRG1-KO and DISC1-LI Mice. *Sci. Rep.* **7**, 42525; doi: 10.1038/srep42525 (2017).

**Publisher's note:** Springer Nature remains neutral with regard to jurisdictional claims in published maps and institutional affiliations.

## Supplementary Material

Supplementary Figures
