# Supplementary material for: Electrical Stimulation Using Conductive Polymer Polypyrrole Counters Reduced Neurite Outgrowth of Primary Prefrontal Cortical Neurons from NRG1-KO and DISC1-LI Mice
Source: Sci Rep. 2017 Feb 15;7:42525. doi: 10.1038/srep42525 (PMC5309772; doi:10.1038/srep42525)
Supplement: Supplementary Figures [file srep42525-s1.pdf]

Electrical Stimulation Using Conductive Polymer Polypyrrole Counters Reduced Neurite Outgrowth of Primary Prefrontal Cortical Neurons from NRG1-KO and DISC1-LI Mice

Qingsheng Zhang, Dorna Esrafilzadeh, Jeremy M. Crook, Robert Kapsa, Elise M. Stewart, Eva Tomaskovic-Crook, Gordon G. Wallace, Xu-Feng Huang

Supplementary Figure S1

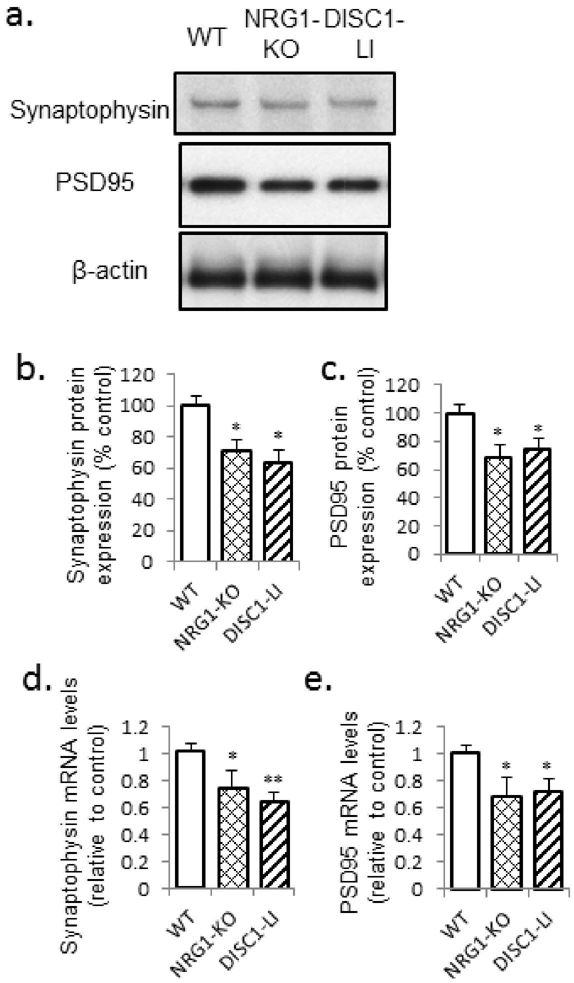

Supplementary Figure S1. NRG1-KO or DISC1-LI inhibits expressions of synaptophysin and PSD95 in mice primary PFC neuronal cultures. (a-c) Protein expressions of synaptophysin and PSD95 are down-regulated in primary PFC neuronal cultures from NRG1-KO and DISC1-LI mice compared to wild-type. (d-e) Synaptophysin and PSD95 mRNA expressions are also downregulated in primary PFC neuronal cultures from NRG1-KO and DISC1-LI mice compared to wild-type. *$p<0.05$ vs wild-type; **$p<0.01$ vs wild-type. Error bars indicate SEM.

Supplementary Figure S2

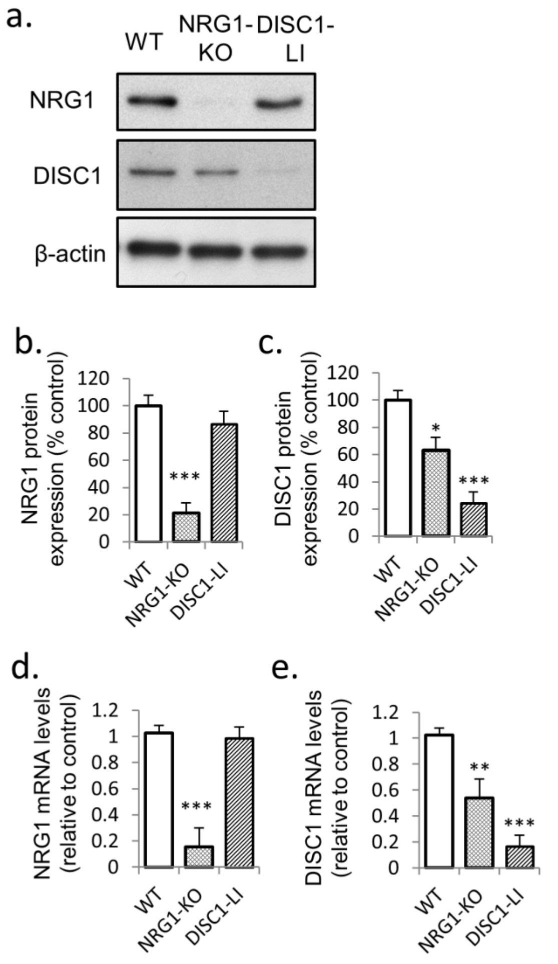

Supplementary Figure S2. Protein and mRNA expressions of NRG1 and DISC1 are altered in neurons cultured from NRG1-KO and DISC1-LI mice. (a-c) NRG1 protein expression is reduced in NRG1-KO model, but not DISC1-KO model. DISC1 protein expression is reduced in both NRG1-KO and DISC1-LI models. (d-e)) NRG1 mRNA expression is reduced in NRG1-KO model, but not DISC1-LI model. DISC1 mRNA expression is reduced in both NRG1-KO and DISC1-LI models. *p<0.05 vs wild-type; **p<0.01 vs wild-type; ***p<0.001 vs wild-type. Error bars indicate SEM.

Supplementary Figure S3

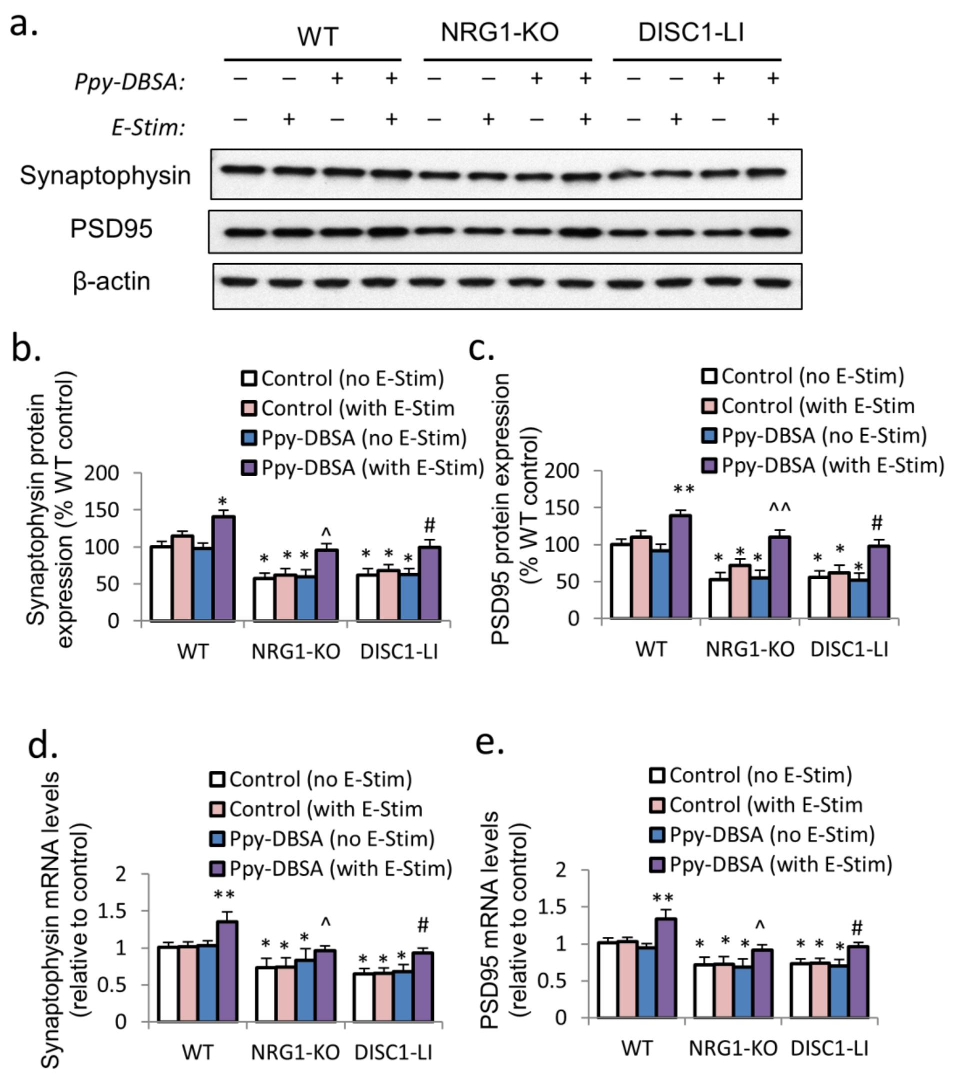

Supplementary Figure S3. Ppy-DBSA with electrical stimulation reversed the reduced expression of synaptophysin and PSD95 in primary PFC neuronal cultures. (a-c) Protein expression of synaptophysin and PSD95 is recovered by electroactive Ppy-DBSA in primary PFC neuronal cultures from NRG1-KO or DISC1-LI. (d-e) mRNA expression of synaptophysin and PSD95 is recovered by Ppy-DBSA in primary PFC neuronal cultures from NRG1-KO or DISC1-LI. *p<0.05 vs wild-type, control; **p<0.01 vs wild-type, control; ^p<0.05 vs NRG1-KO, control; ^^p<0.05 vs NRG1-KO, control; #p<0.05 vs DISC1-LI, control. Error bars indicate SEM.

Supplementary Figure S4

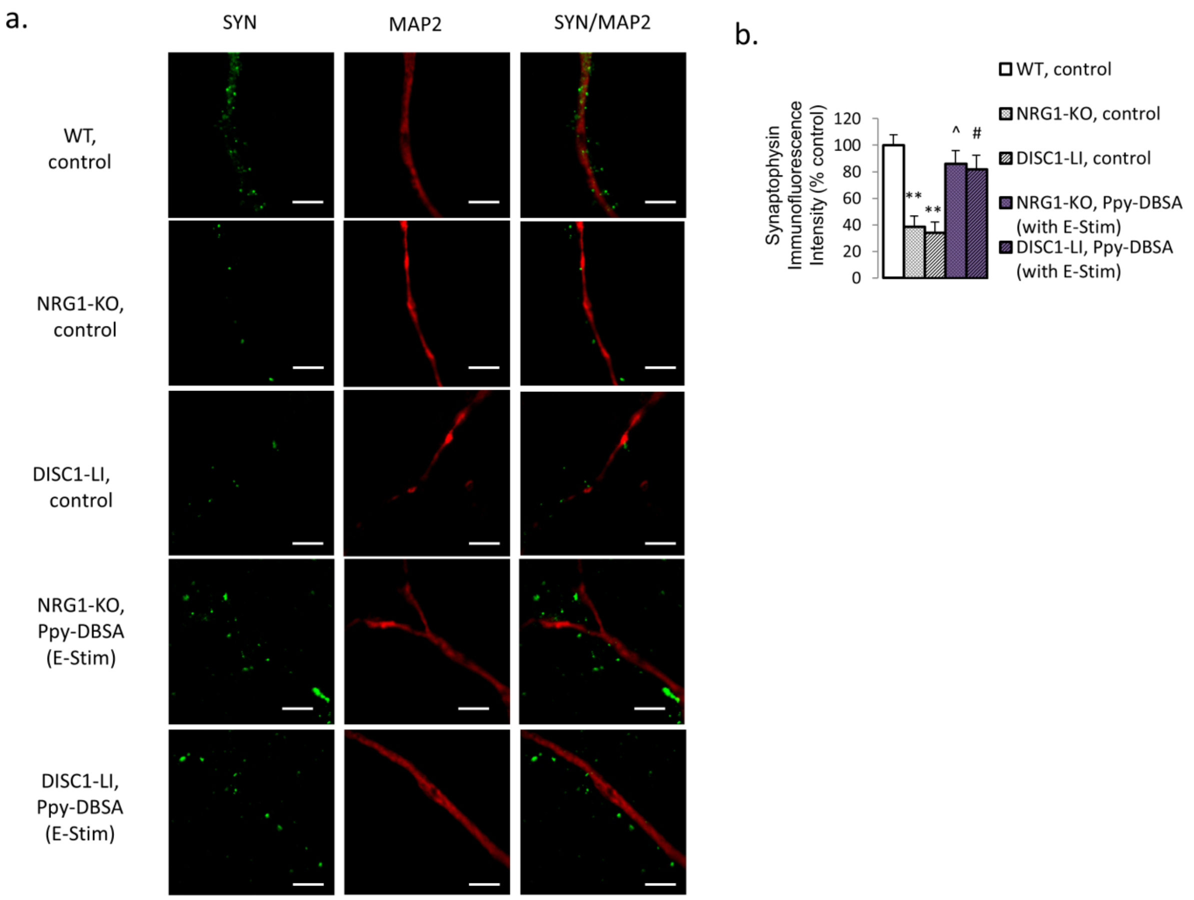

Supplementary Figure S4. Immunofluorescence of synaptophysin is reduced in NRG1-KO and DISC1-LI primary PFC neuronal cultures, but rescued by Ppy-DBSA with electrical stimulation. (a-b) Ppy-DBSA with electrical stimulation reversed the reduced synaptophysin immunofluorescence induced by NRG1-KO or DISC1-LI, in primary PFC neuronal cultures. Scale bar = 5µm. **$p<0.01$ vs wild-type, control; ^$p<0.05$ vs NRG1-KO, control; #$p<0.05$ vs DISC1-LI, control. Error bars indicate SEM.

Supplementary Figure S5

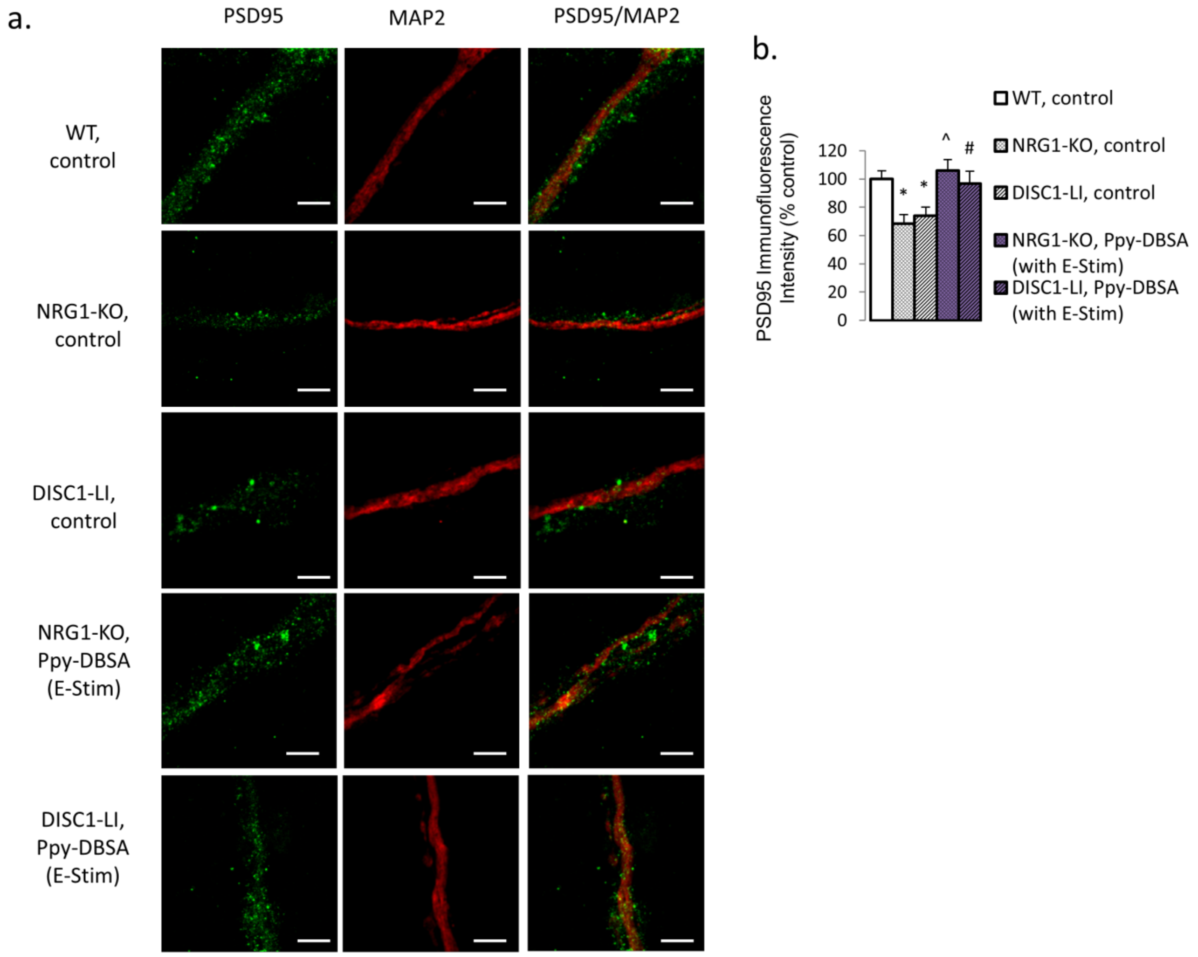

Supplementary Figure S5. Immunofluorescence of PSD95 is reduced in NRG1-KO and DISC1-LI primary PFC neuronal cultures, but rescued by Ppy-DBSA with electrical stimulation. (a-b) Ppy-DBSA with electrical stimulation reversed the reduced PSD95 immunofluorescence induced by NRG1-KO or DISC1-LI, in primary PFC neuronal cultures. Scale bar = 5μm. *$p<0.05$ vs wild-type, control; ^$p<0.05$ vs NRG1-KO, control; #$p<0.05$ vs DISC1-LI, control. Error bars indicate SEM.

Supplementary Figure S6

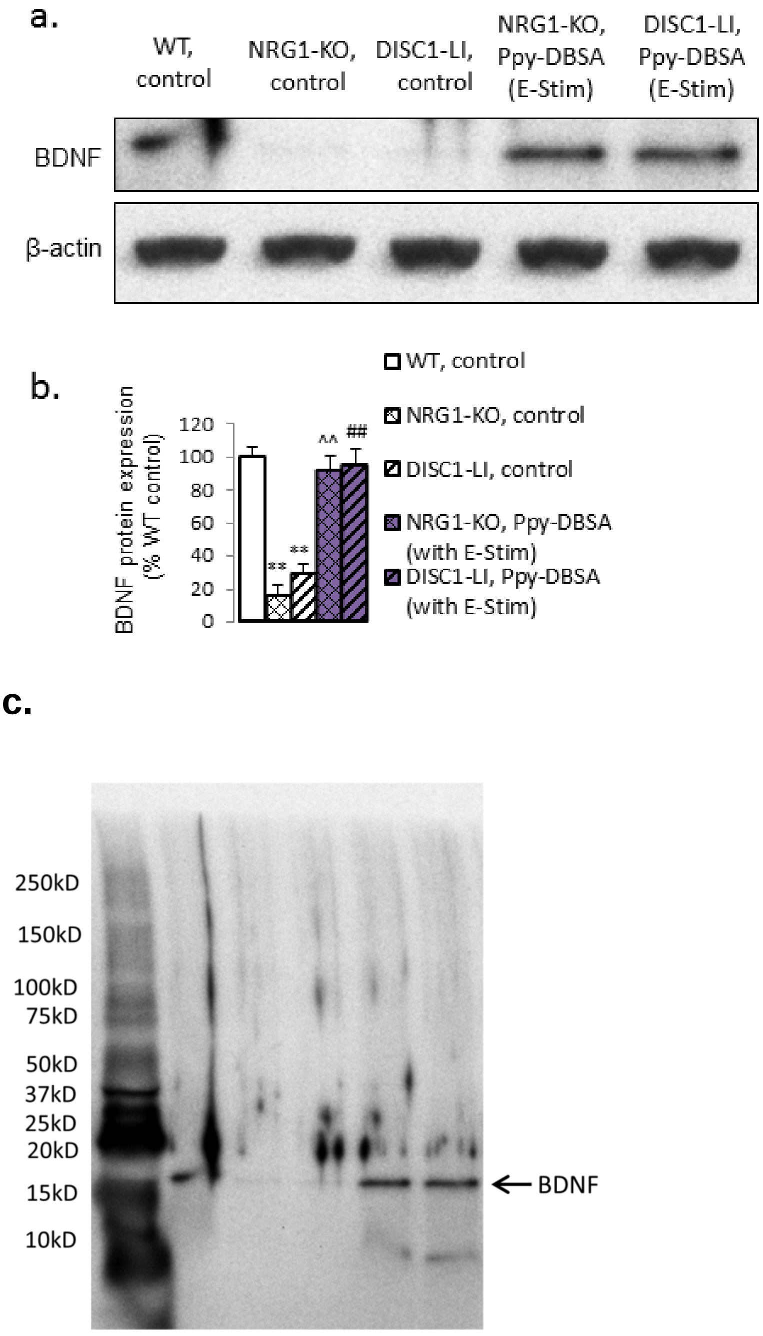

Supplementary Figure S6. Protein expression of BDNF is rescued by electroactive Ppy-DBSA. (a-b) Protein expression of BDNF is rescued by electroactive Ppy-DBSA in primary PFC neuronal cultures from NRG1-KO or DISC1-LI. (c) Whole gel image of BDNF western blotting. *p<0.05 vs wild-type, control; ^^p<0.01 vs NRG1-KO, control; #p<0.05 vs DISC1-LI, control. Error bars indicate SEM.

Supplementary Figure S7

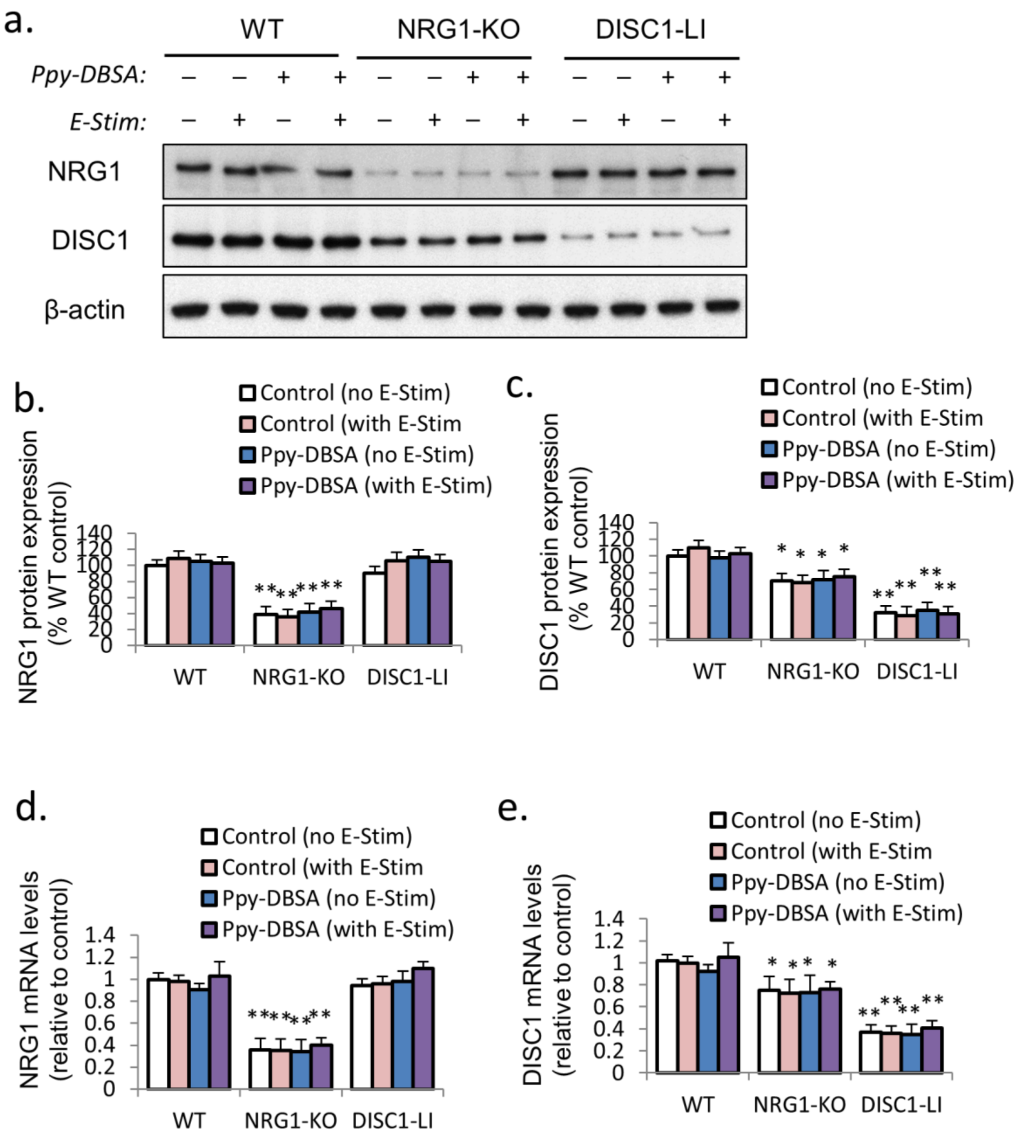

Supplementary Figure S7. Ppy-DBSA or electrical stimulation has no effect on NRG1 or DISC1 expression. (a-c) Ppy-DBSA or electrical stimulation has no effect on protein expression of NRG1 or DISC1, although their expressions are altered in NRG1-KO and DISC1-LI models. (d-e) Ppy-DBSA or electrical stimulation has no effect on mRNA expression of NRG1 or DISC1, although their expressions are altered in NRG1-KO and DISC1-LI models. *p<0.05 vs wild-type, control; **p<0.01 vs wild-type, control. Error bars indicate SEM.

Supplementary Figure S8

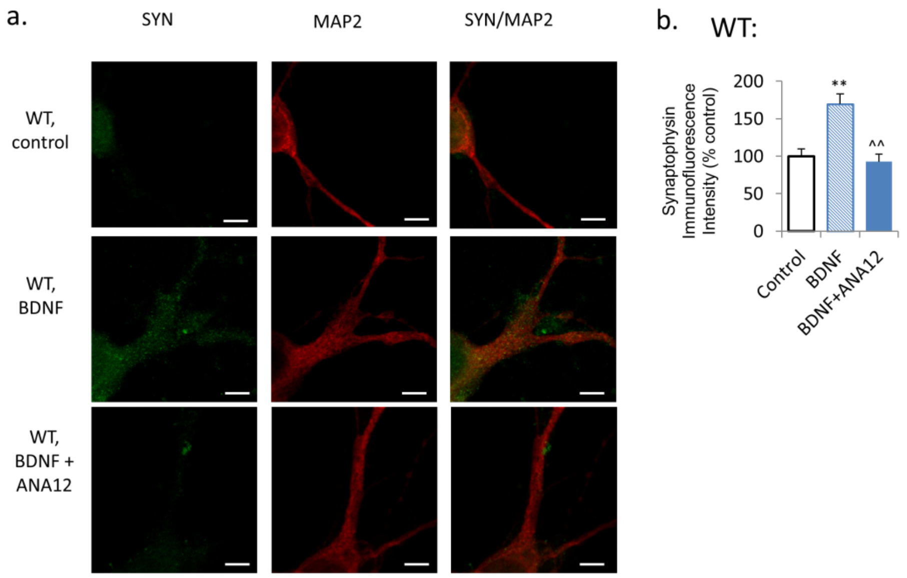

Supplementary Figure S8. Exogenous BDNF improved synaptophysin immunofluorescence in wildtype PFC neurons. (a-b) Immunofluorescence of synaptophysin is elevated by exogenous BDNF treatment, which is blocked by TrkB receptor antagonist ANA12. Scale bar = 5µm. ***p*<0.01 vs control; ^^ *p*<0.01 vs exogenous BDNF. Error bars indicate SEM.

Supplementary Figure S9

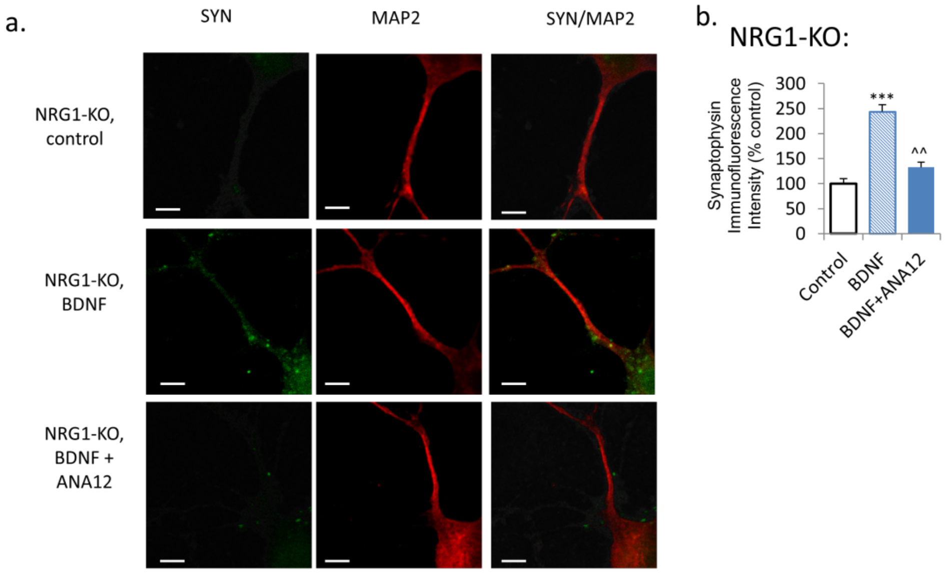

Supplementary Figure S9. Exogenous BDNF improved synaptophysin immunofluorescence in NRG1-KO PFC neurons. (a-b) Immunofluorescence of synaptophysin in NRG1-KO PFC neurons is improved by exogenous BDNF treatment, which is blocked by TrkB receptor antagonist ANA12. Scale bar = 5µm. ****p*<0.001 vs control; ^^ *p*<0.01 vs exogenous BDNF. Error bars indicate SEM.

Supplementary Figure S10

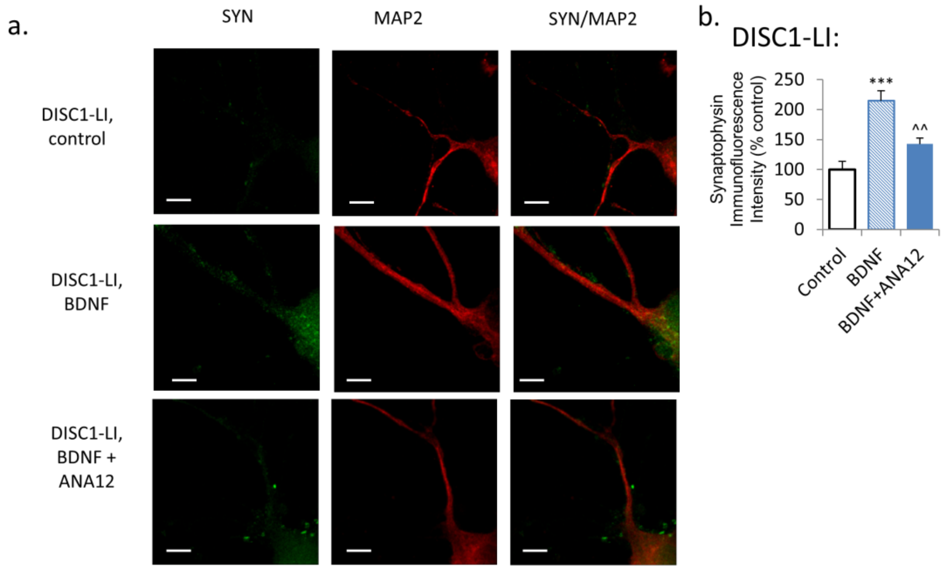

Supplementary Figure S10. Exogenous BDNF improved synaptophysin immunofluorescence in DISC1-LI PFC neurons. (a-b) Immunofluorescence of synaptophysin in DISC1-LI PFC neurons is improved by exogenous BDNF treatment, which is blocked by TrkB receptor antagonist ANA12. Scale bar = 5µm. ***p<0.001 vs control; ^^ p<0.01 vs exogenous BDNF. Error bars indicate SEM.

Supplementary Figure S11

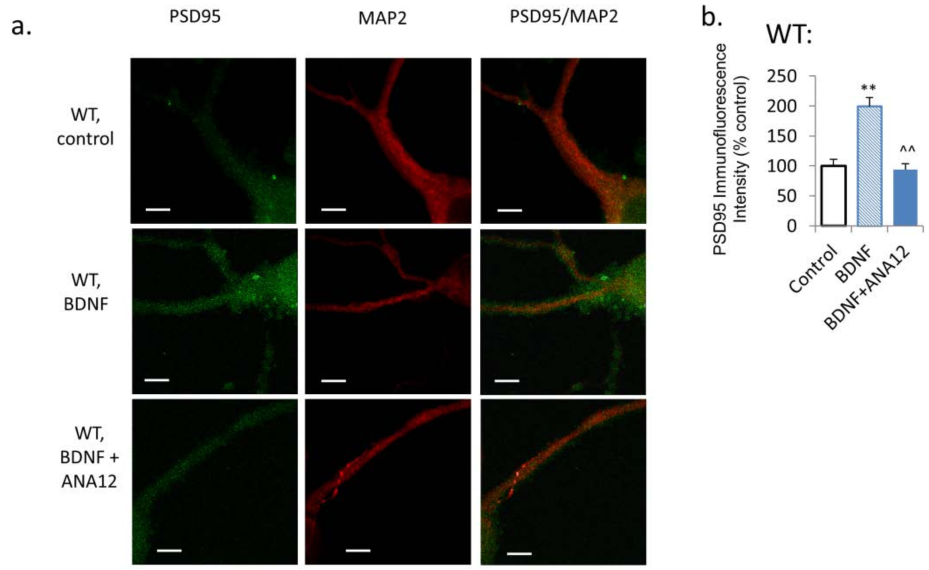

Supplementary Figure S11. Exogenous BDNF improved PSD95 immunofluorescence in wildtype PFC neurons. (a-b) Immunofluorescence of PSD95 is elevated by exogenous BDNF treatment, which is blocked by TrkB receptor antagonist ANA12. Scale bar = 5µm. **p<0.01 vs control; ^^ p<0.01 vs exogenous BDNF. Error bars indicate SEM.

Supplementary Figure S12

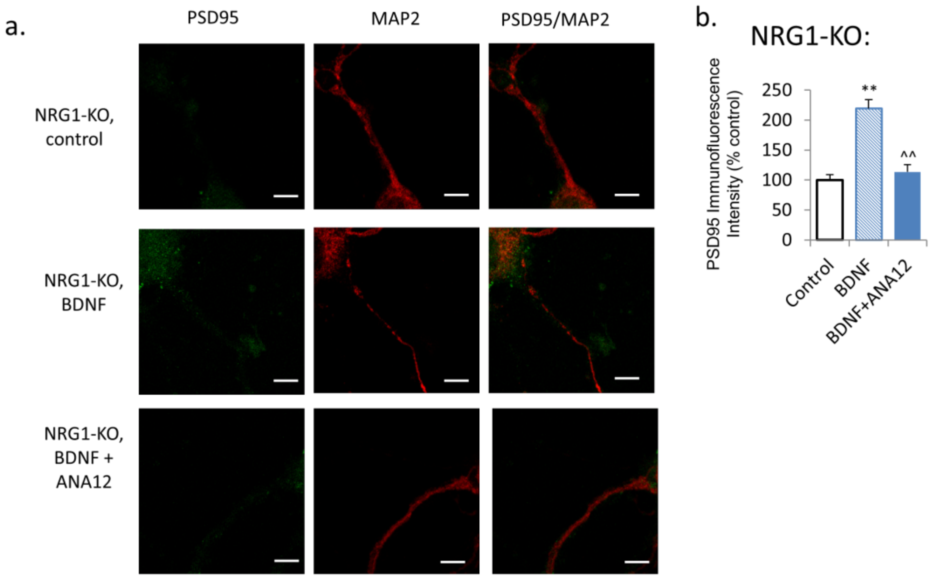

Supplementary Figure S12. Exogenous BDNF improved PSD95 immunofluorescence in NRG1-KO PFC neurons. (a-b) Immunofluorescence of PSD95 in NRG1-KO PFC neurons is improved by exogenous BDNF treatment, which is blocked by TrkB receptor antagonist ANA12. Scale bar = 5µm. $^{**}p<0.01$ vs control; ^^ $p<0.01$ vs exogenous BDNF. Error bars indicate SEM.

Supplementary Figure S13

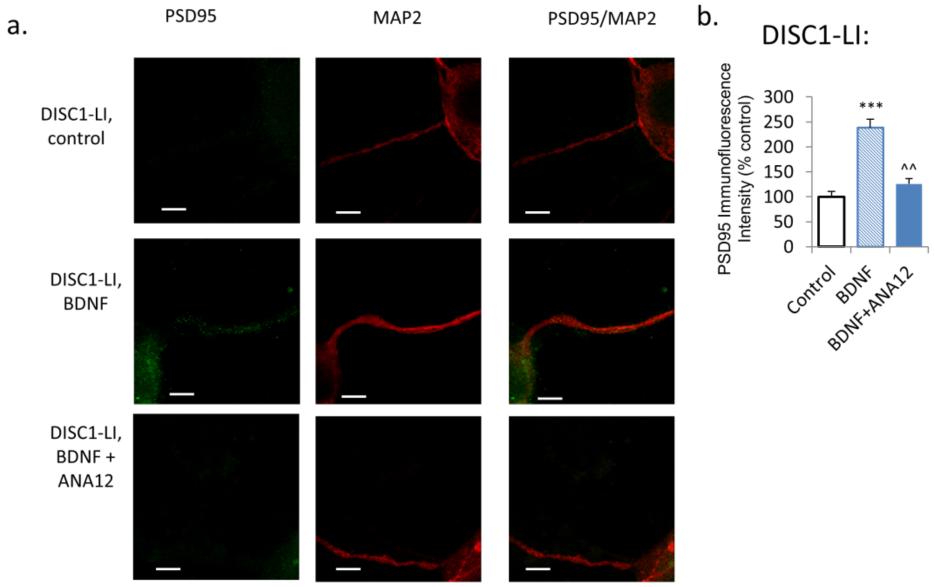

Supplementary Figure S13. Exogenous BDNF improved PSD95 immunofluorescence in DISC1-LI PFC neurons. (a-b) Immunofluorescence of PSD95 in DISC1-LI PFC neurons is improved by exogenous BDNF treatment, which is blocked by TrkB receptor antagonist ANA12. Scale bar = 5µm. $^{***}p<0.001$ vs control; ^^ $p<0.01$ vs exogenous BDNF. Error bars indicate SEM.

Supplementary Figure S14

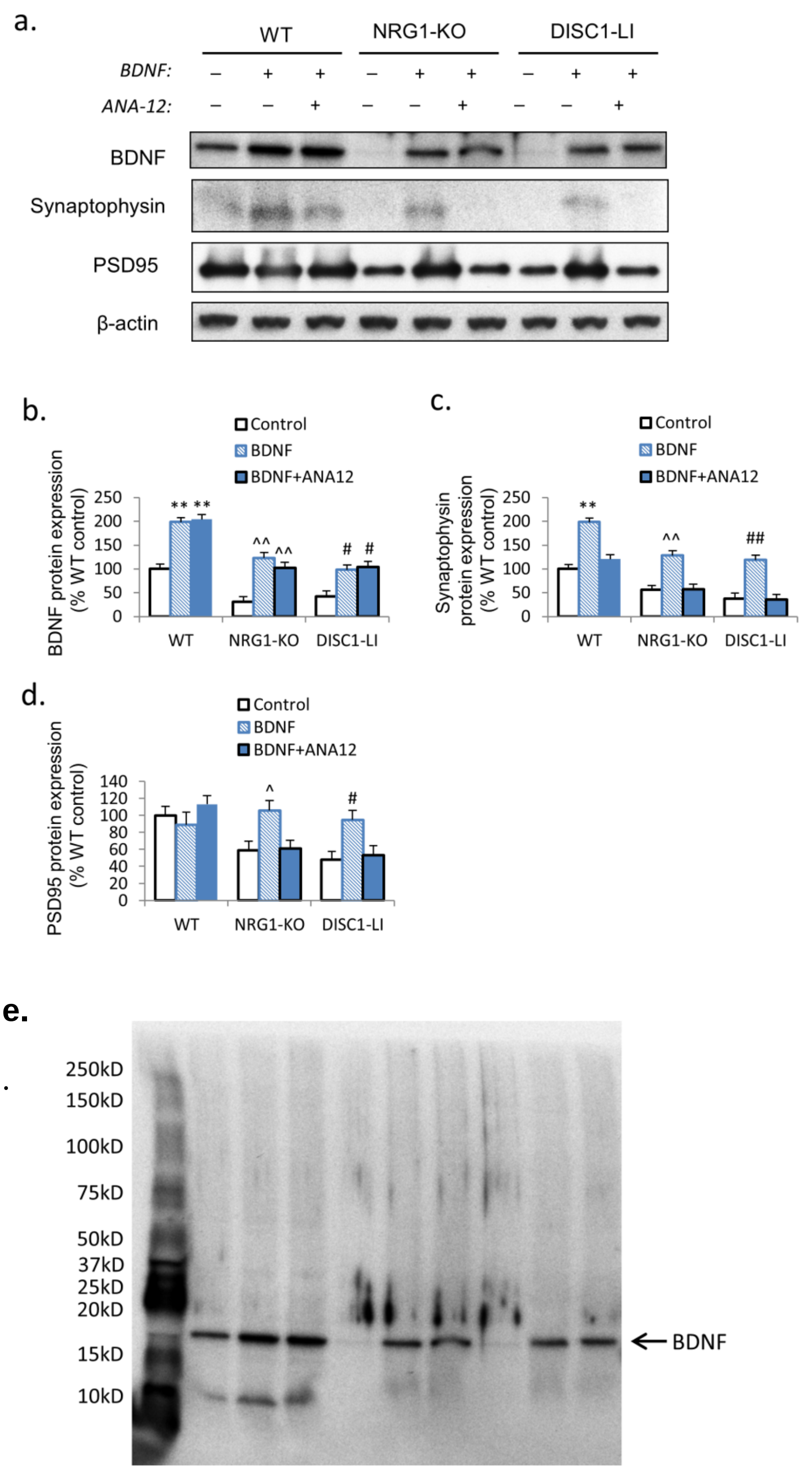

Supplementary Figure S14. Exogenous BDNF improved BDNF, synaptophysin, and PSD95 protein expression in wildtype, NRG1-KO, and DISC1-LI PFC neurons. (a-b) BDNF protein expression is elevated by exogenous BDNF treatment in wildtype, NRG1-KO and DISC1-LI PFC neurons, regardless of co-treatment with TrkB receptor antagonist ANA12. (a; c) Synaptophysin protein expression is improved by exogenous BDNF treatment, which is blocked by TrkB receptor antagonist ANA12. (a; d) PSD95 protein expression is improved by exogenous BDNF treatment in NRG1-KO and DISC1-LI PFC neurons, which is blocked by TrkB receptor antagonist ANA12. (e) Whole gel image of BDNF western blotting.

**$p<0.01$ vs wildtype control; ^ $p<0.05$ vs NRG1-KO control; ^^ $p<0.01$ vs NRG1-KO control; # $p<0.05$ vs DISC1-LI control; ## $p<0.01$ vs DISC1-LI control. Error bars indicate SEM.
